# Theory of Electric Resonance in the Neocortical Apical Dendrite

**DOI:** 10.1371/journal.pone.0023412

**Published:** 2011-08-10

**Authors:** Ray S. Kasevich, David LaBerge

**Affiliations:** 1 Stanley Laboratory of Electrical Physics, Great Barrington, Massachusetts, United States of America; 2 Department of Cognitive Sciences, University of California Irvine, Irvine, California, United States of America; Tel Aviv University, Israel

## Abstract

Pyramidal neurons of the neocortex display a wide range of synchronous EEG rhythms, which arise from electric activity along the apical dendrites of neocortical pyramidal neurons. Here we present a theoretical description of oscillation frequency profiles along apical dendrites which exhibit resonance frequencies in the range of 10 to 100 Hz. The apical dendrite is modeled as a leaky coaxial cable coated with a dielectric, in which a series of compartments act as coupled electric circuits that gradually narrow the resonance profile. The tuning of the peak frequency is assumed to be controlled by the average amplitude of voltage-gated outward currents, which in turn are regulated by the subthreshold noise in the thousands of synaptic spines that are continuously bombarded by local circuits. The results of simulations confirmed the ability of the model both to tune the peak frequency in the 10–100 Hz range and to gradually narrow the resonance profile. Considerable additional narrowing of the resonance profile is provided by repeated looping through the apical dendrite via the corticothalamocortical circuit, which reduced the width of each resonance curve (at half-maximum) to approximately 1 Hz. Synaptic noise in the neural circuit is discussed in relation to the ways it can influence the narrowing process.

## Introduction

The pyramidal neuron, with its relatively long dendrite that extends upward from the top of the soma, is the main excitatory neuron and most numerous type of neuron found in the neocortex [1]. Its electrical activity is influenced by the thousands of other neurons, both excitatory and inhibitory, which make synaptic contacts along the membrane surface [2], [3]. In the waking state, a large proportion of these synaptic contacts are continuously bombarded by local circuits, which maintains a low level of noisy subthreshold oscillations in the neural membrane [4], [5].

Electrical brain rhythms, particularly synchronized oscillations generated by the pyramidal neurons, are measured as encephalograms (EEGs), and are believed to be related to a wide variety of cognitive functions [6], [7]. The mechanisms of these synchronized oscillations have been studied under conditions in which the dendrite membrane voltage lies below the threshold for generating action potentials. Under these subthreshold conditions, an injected current typically induces the neuron to respond by transferring voltage along the dendritic membrane, and in some neurons, the magnitude of this response is frequency dependent. When the neural membrane responds preferentially to inputs within a narrow range of frequencies, the membrane is said to be exhibiting resonance [8], [9]. As it is most generally used, the term *resonance* denotes the ability of a system to oscillate most strongly at a particular frequency.

Resonance activity apparently can arise from noise in neural networks in a variety of ways, ranging from the stochastic resonance produced by a moderate level of synaptic noise that enhances subthreshold signals, e.g., [10], to the coherent resonance produced by combining synaptic noise with network coupling, e.g., [11]. Resonance activity also arises from synaptic plasticity, for example in the regulation by synaptic plasticity of thalamocortical connections of gamma (30–100 Hz) oscillations in the primary visual cortex [12] and in filtering by short term depression and facilitation at synapses [13], [14]. Plasticity has been combined with a coupling of 100 neurons to produce transient stochastic resonance [15]. The present approach regards resonance activity in the neocortical apical dendrite as arising from repeated operations of electrical circuit elements of the apical dendrite membrane (mostly capacitive) on current pulse trains injected into the distal segment of the apical dendrite. These pulse trains arise from the thalamus, and are sustained over time by the corticothalamic loop.

Apparently, transient alterations in synaptic function on the dendrites of the waking animal are continually being initiated by local circuit background activity on dendritic spines, which produce subthreshold membrane activity. Alterations are produced by locally produced action potentials and backpropagating action potentials. The finding [13], [14] that temporal filtering of synaptic transmission arises from short-term synaptic depression and facilitation following synaptic events indicates that recent synaptic activity produces a transient context, and this context can affect local signal processing [16], and perhaps can affect the more global tuning of resonance at the neural or circuit levels.

The purpose of the present paper is to increase our understanding of the mechanisms by which the dendritic membrane can become tuned to a specific narrow band of frequencies by describing a model of the transfer of electrical energy along the apical dendrite of a neocortical pyramidal neuron. As current pulses move down the apical dendrite, they are influenced by the conductive and capacitive elements of the anisotropic membrane (in which capacitance is not the same in radial and longitudinal directions). The progressive narrowing of resonance in the present model of the neocortical apical dendrite is based on the surface impedance theory formulated by Delogne [17]. A standard measurement for describing the electrical properties of the membrane is impedance, which is the ratio of the voltage output to the current input [8], [18]. The relationship of impedance amplitude to resonance characteristics has been investigated in several articles [[8], [19]–[23]. The relationship of impedance phase to membrane resonance has received considerably less attention in the literature, but a recent study [18] highlights the potential role of membrane channel activity on time delays in hippocampal neurons. For purposes of analyzing possible determinants of resonance in the apical dendrite of the neocortical pyramidal neuron, the present study will use impedance amplitude as the principal measurement. In the particular circuit model of this paper, a peak in the transfer impedance amplitude, measured here over the frequency range 10 to 100 Hz, indicates the presence of resonance in the apical dendrite membrane [8]. We will investigate the characteristics of resonance tuning at four frequencies: 20, 40, 60, and 80 Hz.

## Results

### Simulated Model

**Figure 1:**
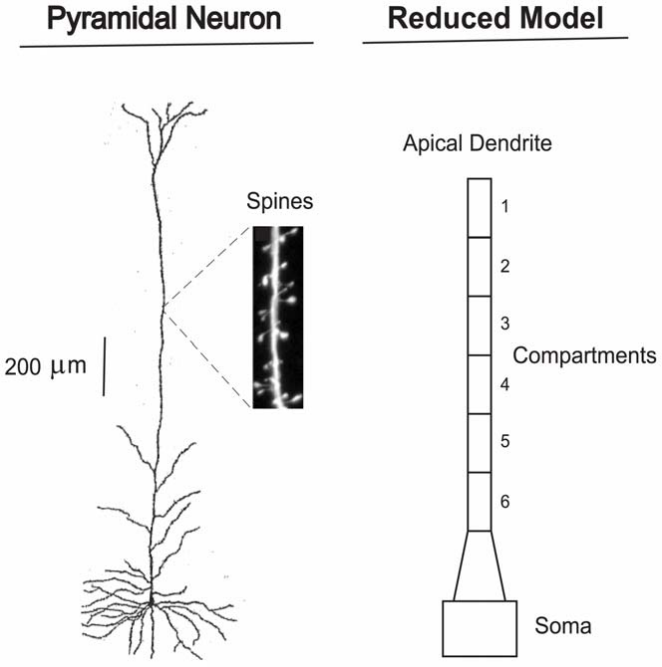
A neocortical layer 5 pyramidal neuron: camera lucida drawing and reduced compartment model. Human Layer 5 neocortical pyramidal neuron from [32], and a simplified compartment model of the apical dendrite. Also shown, on a 18-micron length of dendrite, is a sample from the hundreds of spines that dot the apical dendrite [33] (Figure 8). The schematic drawing of the apical dendrite in the reduced model contains 6 compartments, which provide the present model framework. Between the 6th compartment and the soma is a segment of the apical dendrite that contains distributions of electrical elements that differ from those in the 6 compartments, and this segment is regarded as a transitional region.

The vertical shaft of the typical human neocortical apical dendrite is modeled here as a series of 6 compartments, as shown in Figure 1, following the reconstruction of the apical dendrite by [24]. The 6 compartments constitute the mid region of the apical dendrite, and these 6 compartments, each of a length of 200 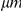, will serve as the main compartment model. An additional dendritic segment, located between the 6th compartment and the soma, has a length here of approximately 240 microns, and may be regarded as a transitional region. The diameter of this segment gradually decreases to accommodate the large difference in diameters of the soma and the mid region of the dendrite, while the 6 compartments maintain a relatively constant diameter [25]. Therefore the present model does not include the transitional segment between the soma and the 6 compartments. The synapses on the initial 40 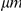 part of the transitional segment, like the soma, are inhibitory and excitatory spines are absent [3]. As the distance from the soma increases the inhibitory synapses decrease in number while the spines increase in number. At the distance from the soma where the 6 model compartments begin, the synapses are mostly excitatory [26]. Membrane conductances are known to fluctuate somewhat over the middle section of the apical dendrite shaft, e.g., [27]–[29]. One of these changes is incorporated into the model, while other changes are tentatively assumed to produce relatively small perturbations in the behavior of the model. In some pyramidal neurons the main shaft of the neocortical apical dendrite bifurcates into two shafts (and in some it bifurcates more than once). The voltage output from each branch will depend on the transfer impedance of that branch and its input conductance. The input voltage of Êeach branch is identical and is derived from the input current at the initial segment multiplied by the segments transfer impedance. When the ends of the branches converge to a common segment leading to the soma, the total voltage experienced by the common branch may combine in a manner that depends on the transfer impedance of each branch. The transfer impedance of a branch will, in turn depend on the number of compartments defined for each branch.

The input from the thalamic principal neuron to the first compartment is located at the distal region of the Layer 5 apical dendrite for all neocortical minicolumns except for minicolumns located in the primary sensory areas, where the input from the thalamic principal neuron is mainly in the mid region of the Layer 5 pyramidal neuron, but with some inputs at the distal region [30], [31]. Relatively thin branches of the apical dendrite that appear most frequently near the soma do not receive direct thalamic inputs, and their exact function in the present model is not specified at this time. It appears that the spines are tonically activated by the same local circuitry that activates spines on the major shafts, and this activation could produce the voltage level needed to open local 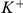
channels in a manner that supports the particular peak frequency resonance of the local minicolumn. In any case, the net contribution of the minor branches is presumed to be relatively weak.

**Figure 2:**
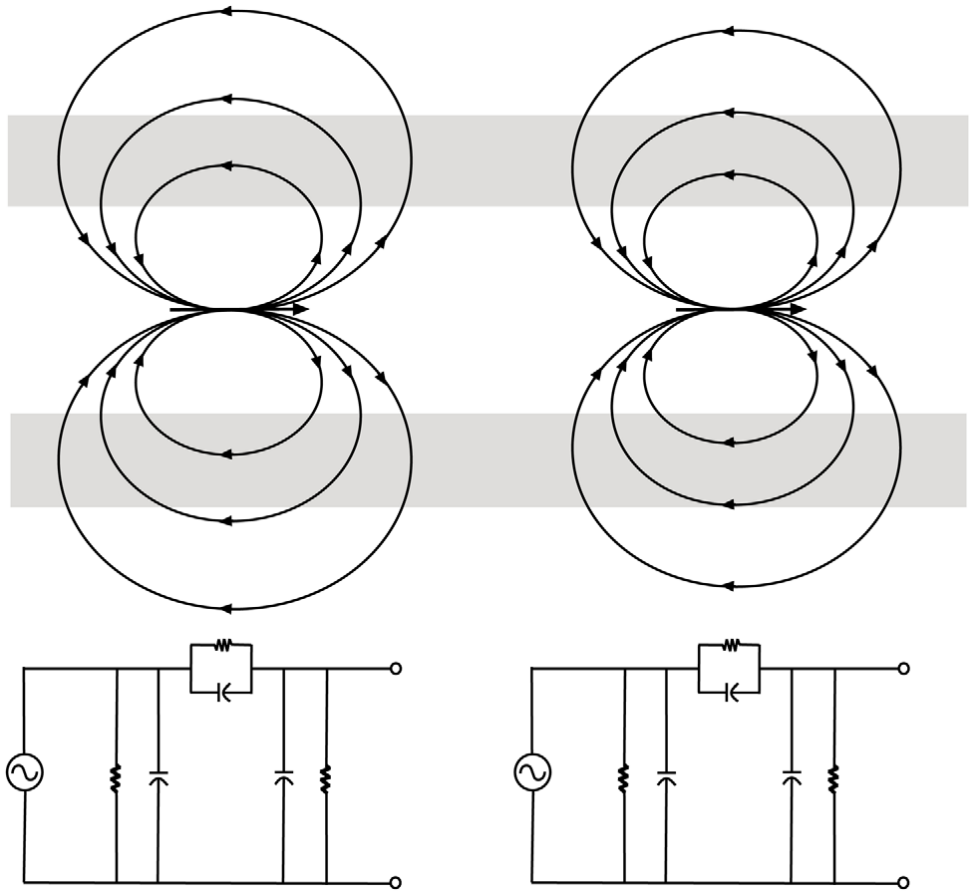
Electric field dipoles and corresponding circuits. Frequency-dependent lumped-element equivalent circuit diagrams of dendritic compartments.

**Figure 3:**
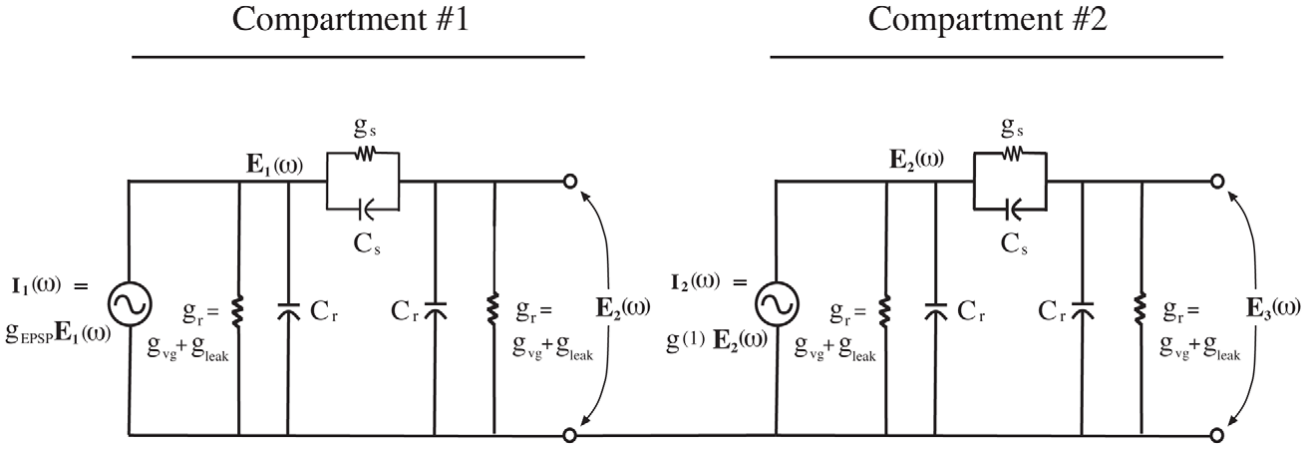
Circuit diagram of two adjacent connected circuit compartments of the membrane. The circuit can be viewed as a two-stage RC coupled amplifier circuit [42].

Figure 2 shows a schematic drawing of a typical longitudinal cross-section of the pyramidal apical dendrite, in which the current-carrying core is surrounded by the lipid bilayer membrane (additional electrical detail of the membrane structure is shown in Figure 3). While the diameter of an apical dendrite has a range of approximately 0.30 to 8.5 microns [34], the thickness of the membrane is considerably smaller, and varies in the range of 3–5 nanometers [35], [36]. Hence the scale of the membrane in Figure 2 is considerably expanded relative to the scale of the cytoplasmic core.

The instantaneous movement of charges is represented in Figure 2 by a dipole arrow. The currents in the dendritic core generate electromagnetic fields, with the electric field being the dominant one [36]. As the energy field travels along the membrane, it acts upon the charges to move them along the axis of the conducting core. This view of the fields as electric dipoles associated with moving charges in a conducting medium follows from Maxwellian concepts, and some of the earliest articulations of this view were given by [37], [38]. The electric fields of the idealized dipole loop outward and back through the membrane dielectric and extracellular fluid to terminate on the dipole, thus completing the circuit. A steady-state frequency lumped-element representation of a neuron compartment suitable for frequency analyses of voltages and currents associated with the moving dipole field is shown in Figure 3.

Each circuit compartment contains an active voltage-gated outward conductance, 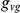, a passive leak conductance, 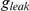, and the capacitances, 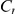 and 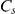, which represent anisotropic membrane electrical properties. The first compartment contains, in addition to these circuit elements, a synaptic input conductance, 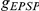. The compartment equivalent circuit is a series-parallel RC circuit with voltage-dependent current sources. The preceding compartment output voltage defines the voltage-dependent input circuit source when multiplied by the compartment input conductance.

The present formulation may be considered a variation of traditional cable theory [39]. Traditional cable theory is based on electromagnetic energy transmission carried by two concentric conductors separated by a dielectric. The inner or center conductor is of positive polarity and carries current and voltage from the source to the end point of the cable or to a load. The outer conductor or shield of negative polarity carries the return current to the source. The shield is assumed to be continuous or uninterrupted. The electric circuit theory of the cable is completely described by series connected resistance (R) and inductance (L) elements, along with shunt connected capacitance (C) and conductance (G), together forming a continuous ladder-like electric network. The presence of inductive components normally enables the high-pass filtering in resonance behavior. The major problem that arises when classical cable theory is applied to neurons is to find a component in the membrane that acts like an inductor. One candidate for an inductive-like component is the temporal activity of h-channels [18]. The time delays in opening and closing of h-channels produce inductive-like effects at frequencies below 20 Hz (and therefore are appropriate for models of hippocampal activity in the 6–12 Hz theta range). However, because the opening and closing of h-channels is not sufficiently fast to account for inductive effects at frequencies above 20 Hz (18), another way must be found that produces high-pass filtering if resonance behavior is to be revealed in the neocortical apical dendrite, which produces EEGs in the 30–100 Hz range, and sometimes higher.

**Figure 4:**
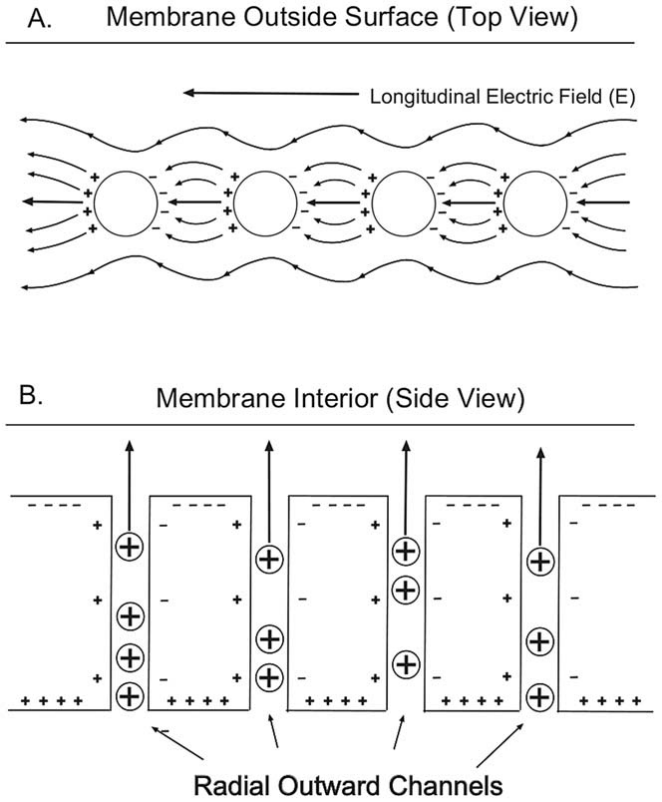
Longitudinal and radial capacitances. (A) Longitudinal surface capacitance, acting across the membrane segments between channel openings. (B) Radial capacitance, acting across the dielectric segments between the intracellular and extracellular charges.

The approach taken here modifies the circuit of the traditional cable model by replacing the inductance (L) element with a capacitive element that is directed along the outer surface, longitudinal to current, so that the circuit now contains two kinds of capacitive elements: the non-linear surface capacitance, 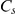, and the radial capacitance, 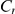, which is the capacitive element in traditional cable theory that is directed perpendicularly across the membrane (see Figure 4). Energy flow along the outside of the membrane activates the longitudinal surface capacitive elements, and this energy flow is produced by holes or apertures in the shield of the traditional cable. The result is a leaky cable with a dielectric coating, which is a variation of the cable model that includes possibilities of external energy flow coupled to internal energy flow. The leaky feature may involve radiation, and is used in many practical applications such as tunnel communications (e.g. in the English Channel Tunnel and the Ted Williams Tunnel in Boston). The external current flows through a surface impedance associated with the boundary between the outer surface of the perforated sheath and external medium which is air for the engineering application and extracellular fluid for the dendritic application. The electromagnetic theory of leaky cables in air was developed by Delogne [17].

Delogne's theory [17] has guided our description of surface current energy flow along the outer membrane of the apical dendrite. The apertures or channels are represented by a nonlinear capacitance and conductance coupled to the internal RC parameters of the cable. Unlike the radial capacitance, the surface capacitance and conductance of the dendrite has a frequency, geometry, and wave number dependence. This new combination of electric circuit elements leads in a straight-forward manner to band-pass frequency behavior for the dendrite.

The apical dendrite model we have proposed has a single peak value in the band-pass characteristic not because of any energy exchanges back and forth between inductance L and capacitance C as in electric circuit theory. A resonance or peak amplitude in the transfer impedance of the neuron is created by the way the electric energy divides between the surface and membrane capacitive and conductive elements as a function of frequency. At low frequencies, capacitance tends to block current flow and at high frequencies capacitance tends to act as a short.

The horizontal capacitance 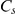 and horizontal conductance 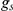 are derived from the surface wave electrical properties of the membrane and extracellular fluid interface. Delogne [17] derived the general surface impedance of the leaky cable with dielectric coating by using electromagnetic field theory. The surface impedance component of the circuit is capacitive in nature owing to dielectric coating of the leaky cable. Surface impedance is a fundamental electrical property of the external energy flow. According to Barlow and Brown (see p. 5 in [40]), “A surface wave is one that propagates along an interface of two different media without radiation; such radiation being construed to mean energy converted from the surface wave field to some other form”. In the present case, the surface wave includes both the instantaneous capacitive stored energy associated with moving charges along the interface between the outer membrane surface and extracellular fluid, represented by 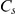, and the dissipation of energy associated primarily with the internal energy loss of the dendritic core and extracellular fluid, represented by 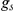. The surface impedance concept provides an anisotropic membrane model of the neuronal dendrite, which in this respect, contrasts with the compartment model of [20].

For the single compartment, the derived steady-state complex transfer impedance is: 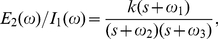
(1)


where 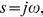
(2)

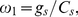
(3)

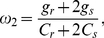
(4)

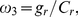
(5)


and
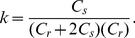
(6)


The parameters, 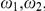 and 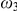 have units of radian frequency and are referred to here as relaxation frequencies, following classical circuit theory concepts. The relaxation frequencies and 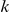 completely characterize the bandpass frequency behavior of the dendrite model.

Equation (1) contains expressions for two different capacitances: 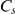, and 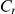, corresponding, respectively, to the holding of charge in the longitudinal direction of the membrane and the holding of charge in the radial direction of the membrane. The operation of 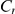 is crucial for producing the low-pass filtering at frequencies below the peak frequency, while the operation of 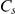 is crucial for producing the high-pass filtering at frequencies above the peak frequency.

The dynamic characteristics of this network model can be specified by either its voltage transfer function, 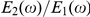, or its impedance transfer function, 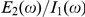. Equation (1) is the typical form for the frequency response of an RC coupled electronic amplifier with bandpass behavior governed by frequency, 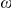, and the values of 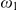, 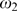, 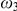, and constant 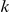 [41].

The sheath geometry for the classical leaky cable involves a cylindrical outer surface defined by a two-layer construction: a conducting layer on one side and a dielectric layer on the other side. Holes (channels) exist through the surface allowing current flow from inside the cable to outside or in the opposite direction. The surface impedance representation of the current and voltage boundary condition for a leaky cable is assumed to be approximately equivalent to the dendrite outer surface impedance boundary in the range of neuronal resonant frequencies of our paper by symmetry. The effect of attenuation or resistivity (conductivity) in Equation (7), is given by the propagation constant or wave number for the classical neuron cable model [36]. The ratio of the longitudinal electric field E to the circumferential magnetic field H evaluated at the boundary separating conductor and dielectric gives the result of Delogne's [17] surface admittance as shown in Equation (7), separated into capacitive and conductive components.

The surface transfer admittance derived from the Delogne leaky cable theory [17] is approximately
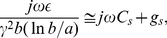
(7)


where 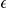 is the membrane dielectric constant, 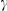 is the complex propagation constant given by [36], 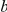 is the outer radius of the membrane, and 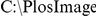 is the inner radius of the membrane. The membrane dielectric constant includes the conductive channels, as described by [42] in artificial dielectric theory.

Using the definition of the classical cable propagation constant, 
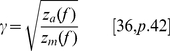
(8)


and the well-known formulas for the intracellular impedance, 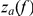, and for the membrane impedance, 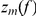, the horizontal capacitance, 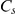, and associated conductance, 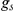, are from Equation (7):
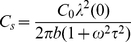
(9)


and
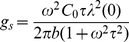
(10)


and
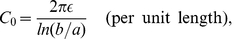
(11)


where 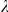 is the neuronal space constant, 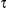 is the neuronal time constant, 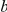 is the outer radius of the membrane, and 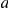 is the inner radius of the membrane.

The electrical theory of leaky cables applied here to the dendritic segment provides a way to consider the effect of coupling between longitudinal currents (along the outer surface of the membrane) and radial membrane currents (across the membrane). If we consider the charge per unit length on each of two concentric cylindrical coaxial capacitor surfaces, the per unit length capacitance of a coaxial cable is given by Equation (11) [43]. The numerator of 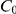 is an expression of area and the denominator is an expression of thickness, which together correspond to the basic physical description of a capacitor. The surface (longitudinal)capacitance, 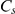, is proportional to 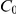, and the parameter of proportionality is dependent on frequency. The radial capacitance, 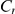, is also proportional to 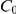, but is not dependent on frequency. If 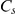 were set to zero (implying that 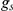 would be changed to represent the core conductance of the dendrite), the present model would reduce to the traditional cable model.

The many radial leak paths of the membrane containing high conductivity fluid greatly increase the membrane conductivity over that of the axon membrane. Although many kinds of conductances exist in the dendritic membrane [44], the present model is based mainly on the outward potassium (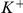) conductances, which are of two kinds: passive leak conductance, 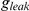 and active voltage-gated conductance 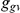. For convenience these two 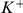 radial conductances are combined under the symbol, 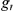. The passive membrane of the dendrite with its radially oriented leak channels constitutes an anisotropic dielectric (see Figure 4). The electrical conductivity in the radial direction is made up of conductive channels with an intracellular resistivity on the order of 29.7 ohm-cm (see p. 481 in [36]). Axon membrane resistivity, in contrast, is on the order of 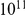 ohm-cm (see p. 483 in [36]). This anisotropic condition strongly influences the shape and location of the membrane model passband as a function of frequency and amplitude.

To observe the operation of the one-compartment circuit over the 10–100 Hz range where most of the EEG measurements in the literature are located, we estimated the transfer impedances that showed peak values at 20, 40, 60, and 80 Hz. Using the circuit parameters assumed for the present model, we obtained maximum impedance amplitude values for these 4 frequency locations of 2.5369, .46009, .13223, and .04952 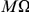, corresponding, respectively, to 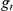 values of 105, 310, 627, and 1056 nS.

### Impedance transfer function for amplitude and phase in the single compartment

**Figure 5:**
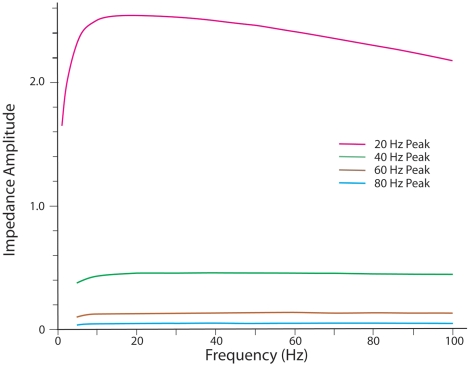
Impedance amplitude curves. Impedance amplitude curves produced from four outward conductances resulting from four levels of internal voltage produced by subthreshold activity of local dendritic spines. Peak amplitudes of the curves are at 20, 40, 60, and 80 Hz.

The four transfer impedance amplitude curves shown in Figure 5 were calculated from Equation (12). Because the expression of Equation (1) involves the imaginary number 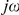, (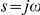), Equation (1) was converted to the real number form:
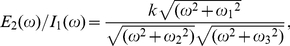
(12)


where 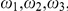 and 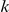 are defined by Equations (3–6).

To illustrate the amplitude calculation of the impedance values shown in Figure 5, we arbitrarily select the 50 Hz point on two curves: the 20 Hz and the 40 Hz peak frequency curves. Entering into Equation (12) the values for 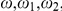 and 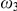 of 314, 2957.88, 1680.82, and 1112.52, respectively, with 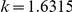
×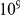,we obtain for the 20 Hz peak frequency curve an impedance of 2.45 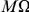 at 50 Hz. For the 40 Hz peak frequency curve we change only the values of 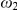 and 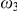 to 3183.98 and 3284.59, respectively, to obtain an impedance of 0.460 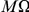 at 50 Hz (see Figure 5).

**Figure 6:**
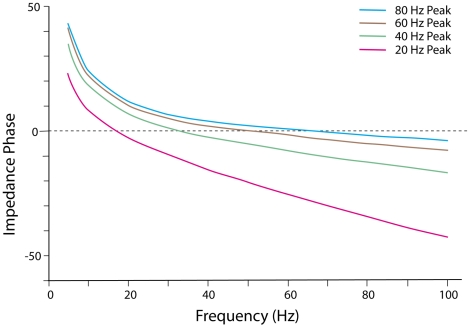
Impedance phase curves. Impedance phase shift curves produced from four outward conductances resulting from four levels of internal voltage produced by subthreshold activity of local dendritic spines.

The corresponding phase angles calculated from Equation (1) are shown in Figure 6. The net phase angle for each compartment derived from Equation (1) is:
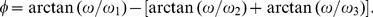
(13)


When we insert into Equation (13) the same 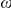 parameter values we just used to calculate the two impedance amplitude points at 50 Hz, we obtain 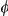 values of 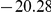 degrees and 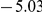 degrees for the 20 Hz and 40 Hz peak frequency curves, respectively (see Figure 6).

The impedance amplitude curves, shown in Figure 6, indicate that current injected into the circuit of one compartment produces a voltage output that shows moderate variation for the 20 Hz peak curve, for which the radial conductance value is relatively low (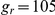 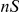). The variation in impedance amplitude shows a slight narrowing around the peak frequency of 20 H (particularly by decreases in amplitude at lower neighboring frequencies), which enables the circuit to pass input current at frequencies near 20 Hz, while exerting a filtering effect on input current at other, more outlying frequencies. However, as the radial conductance increases, and the frequency of the peak impedance amplitude increases with it, the shape of the impedance curve flattens, until, at a peak amplitude frequency of 80 Hz a narrowing of the curve around the 80 Hz peak is no longer detectable by visual inspection. Therefore, the progressive flattening of the curve as radial conductance increases results in a lowering of the ability of the single compartment circuit to operate effectively as a bandpass filter.

The impedance phase curves, shown in Figure 6, depart slightly from resonance phase curves that are based on simple RC-coupled circuits, because the frequencies of the zero-crossings do not exactly correspond to the frequencies of the peak amplitudes for each curve. These deviations at the zero crossings are small for the 20 Hz peak amplitude, and increase toward the 80 Hz peak amplitude. Therefore, the maximum energy transfer for a given set of circuit parameter values occurs at a frequency where the voltage and current are slightly out of phase. An explanation for this deviation is that the longitudinal or coupling capacitance is frequency-dependent in the present circuit, while it is frequency-independent in the simple RC-coupled circuit.

**Figure 7:**
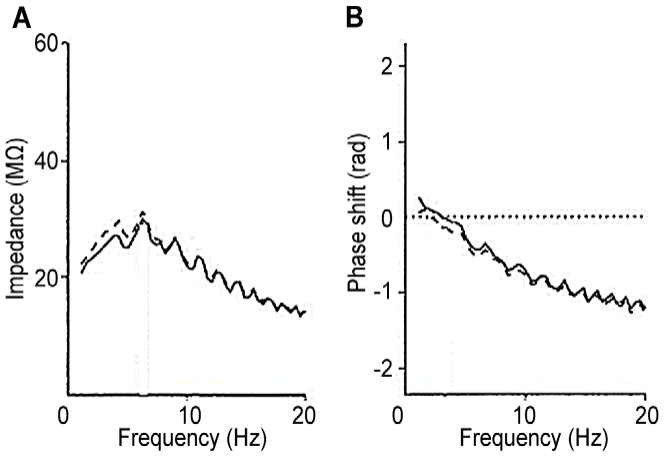
Empirical impedance amplitude and phase curves. (A) Impedance amplitude profile. (B) Impedance phase shift curve. Recording electrode inserted in the soma (filled line), or inserted into the dendrite (dashed line)120–270 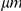 from the soma. Data from [45].

The general characteristics of the impedance amplitude and phase curves of the present model, shown in Figures 5 and 6, can be compared with the empirical curves obtained by an *in vitro* experiment with rats [45], in which chirps of frequencies of 0.1 to 25 Hz were injected into a Layer 5 apical dendrite either at the soma or at distances of 120–270 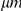 from the soma, while a second electrode recorded the voltage response at the other location. The measured impedance amplitudes and phase are reproduced here in Figure 7. The obtained curves of amplitude and phase shift appear to be independent of the direction of excitation along the apical dendrite. The impedance amplitude curve shows a peak at approximately 6 Hz, and the zero-crossing of the phase at approximately 3 Hz. The maximum impedance level is higher than the level obtained in the present analysis by a factor of approximately 12. This difference in maximum impedance may be due to the lower frequency of the obtained peak resonance and to the passive state of the dendritic membrane in the preparation used by [45].

### Simulated Narrowing of the Resonance Curve

The amplitude curves shown in Figure 5 indicate that the present anisotropic membrane model generates impedance transfer functions that show a peak value at a specific frequency in the range 10–100 Hz. Therefore the transfer functions appear to operate as filters which select a particular frequency or band of frequencies of pulse inputs while blocking other frequencies. However, the effectiveness of selecting or filtering frequencies appears to decrease as the peak frequency of the curves move from 20 to 80 Hz because the slopes of the curves on each side of the peak frequency become progressively smaller. Clearly, the present single compartment circuit model is not adequate for producing effective filtering of input frequencies over the 10–100 Hz range, nor is the model adequate for supporting other activities related to the existence of a sharp resonance at a specific frequency (e.g., [9]).

**Figure 8:**
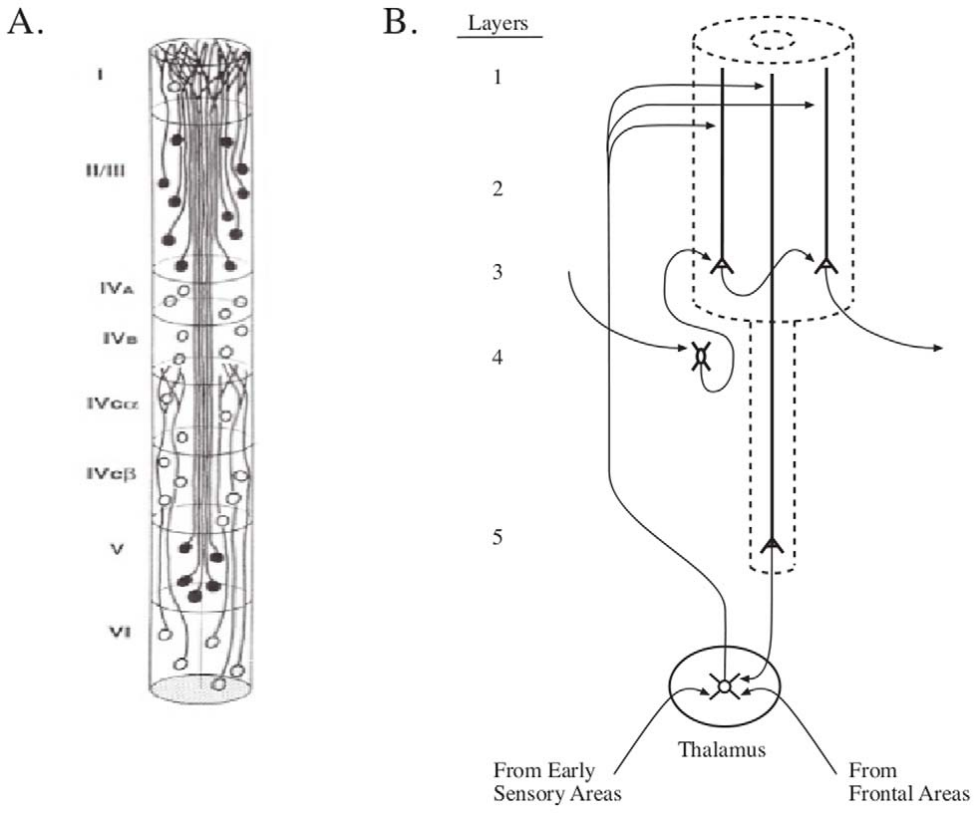
Cortical minicolumn and corticothalamic circuit. (A) Schematic diagram of a minicolumn in monkey visual cortex (see Figure 19 in [46]). Shown are somas and apical dendrites of pyramidal neurons and somas of stellate neurons. (B) Schematic diagram showing the corticothalamic loop. A specific resonance profile in a Layer 5 apical dendrite is projected, via the thalamus, back to the first compartment of the same apical dendrite. The profile is also projected to apical dendrites of Layer 2/3 pyramidal neurons where it spreads to the basal dendrites and may influence the selection of inputs arriving from other cortical minicolumns. For clarity, the reticular nucleus, with its inhibitory projections to the thalamic principal neurons, is not shown. Adapted from [30], [31].

To achieve a narrow, peaked resonance curve, we extend the present single compartment model to a 6-compartment model, with the added feature that the pulse train is recycled repeatedly through the 6 compartments by way of the thalamus within the corticothalamic circuit. Figure 8 shows a schematic diagram of the corticothalamic circuit within which a pulse train repeatedly traverses the compartments of the apical dendrite of the neocortical Layer 5 pyramidal neuron. Each time the pulse train travels through the 6 compartments of the dendrite it produces a cascade of circuit operations which successively narrow the impedance transfer function around its peak value. The thalamus sends current pulses not only to the distal part of the Layer 5 apical dendrite, completing the loop activity, but it also sends current pulses to the distal part of the layer 2/3 apical dendrite, which may influence the input-output activity of the minicolumn within corticocortical circuits that interconnect regions of the cortex [32]. For simplicity in analyzing the general properties of the cascade-loop model, it is assumed initially that no noise is added to the pulse train as it moves from the pyramidal neuron through the thalamus and back to the distal region of the pyramidal neuron. In the Discussion Section the issue of synaptic noise in the apical dendrite spines and in the corticothalamic circuit will be addressed.

**Figure 9:**
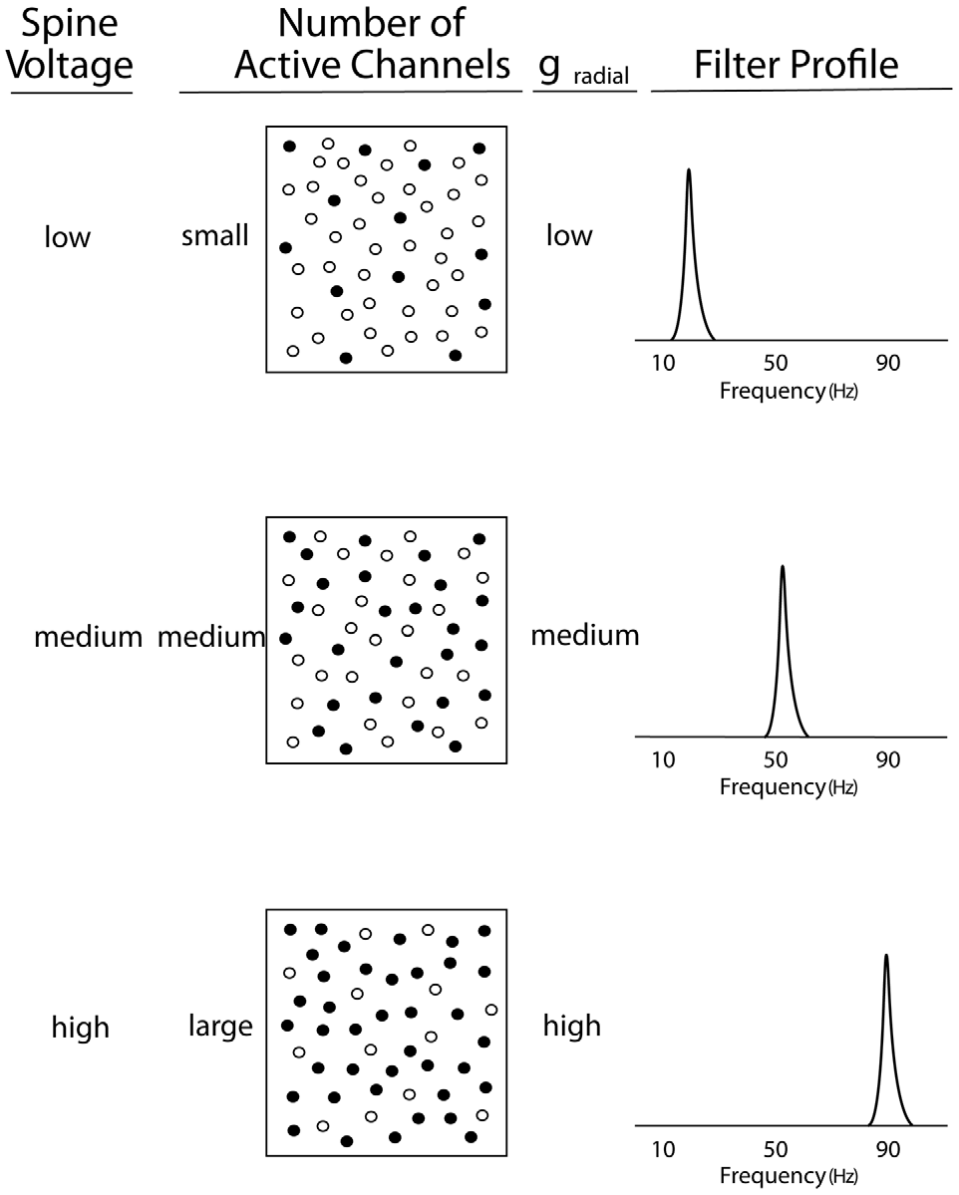
Resonance peak frequency and activity of local spines. Schematic diagram of the hypothesized relationship between peak frequency of a resonance curve and the internal voltage level produced by subthreshold activity of local dendritic spines.

Both the single compartment and the looping multiple-compartment models assume that the average level of the radial conductance, 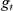, determines the frequency location of the peak in the transfer function. Figure 9 shows, in a local sector of the dendrite, a schematic representation of three levels of voltage-gating which are produced by varying the number of active channels in the membrane. This figure also shows the predicted narrowed shape of the impedance transfer function after a moderate number of loops through the 6 compartments have successively operated on the original current input. It is assumed that the same level of 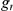 (allowing for slight changes in the leak current for purposes of the model; see Equation 15) operates across all of the compartments of the model, although the level of 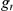 is assumed to vary for apical dendrites across cortical locations.

Although transmembrane ion channels are distributed non-uniformly over a segment of dendritic membrane [27], we assume for simplicity that the present derivation of the radial conductance, 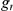, can be reasonably based on averages over the large number of channel openings in a typical segment of the apical dendrite. The activation of the voltage-gated channels is presumed to be produced through the many local spines in a segment of dendrite. This subthreshold activation is, in turn, activated in a tonic manner by the bombardment of local axons, which is commonly termed background noise. Cortical neurons, including the pyramidal neurons, receive a large majority of their synaptic inputs from neurons in local circuits that are less than a millimeter away [2]. The average local bombardment level at a cortical minicolumn is presumed to be constant, on average, but the level is presumed to vary among the minicolumns across the neocortex. Hence, the level of local subthreshold synaptic activation for a cluster of apical dendrites in a particular minicolumn can determine the resonant frequency of those apical dendrites.

### Impedance transfer function magnitude with repeated cascades across the 6 compartments

**Figure 10:**
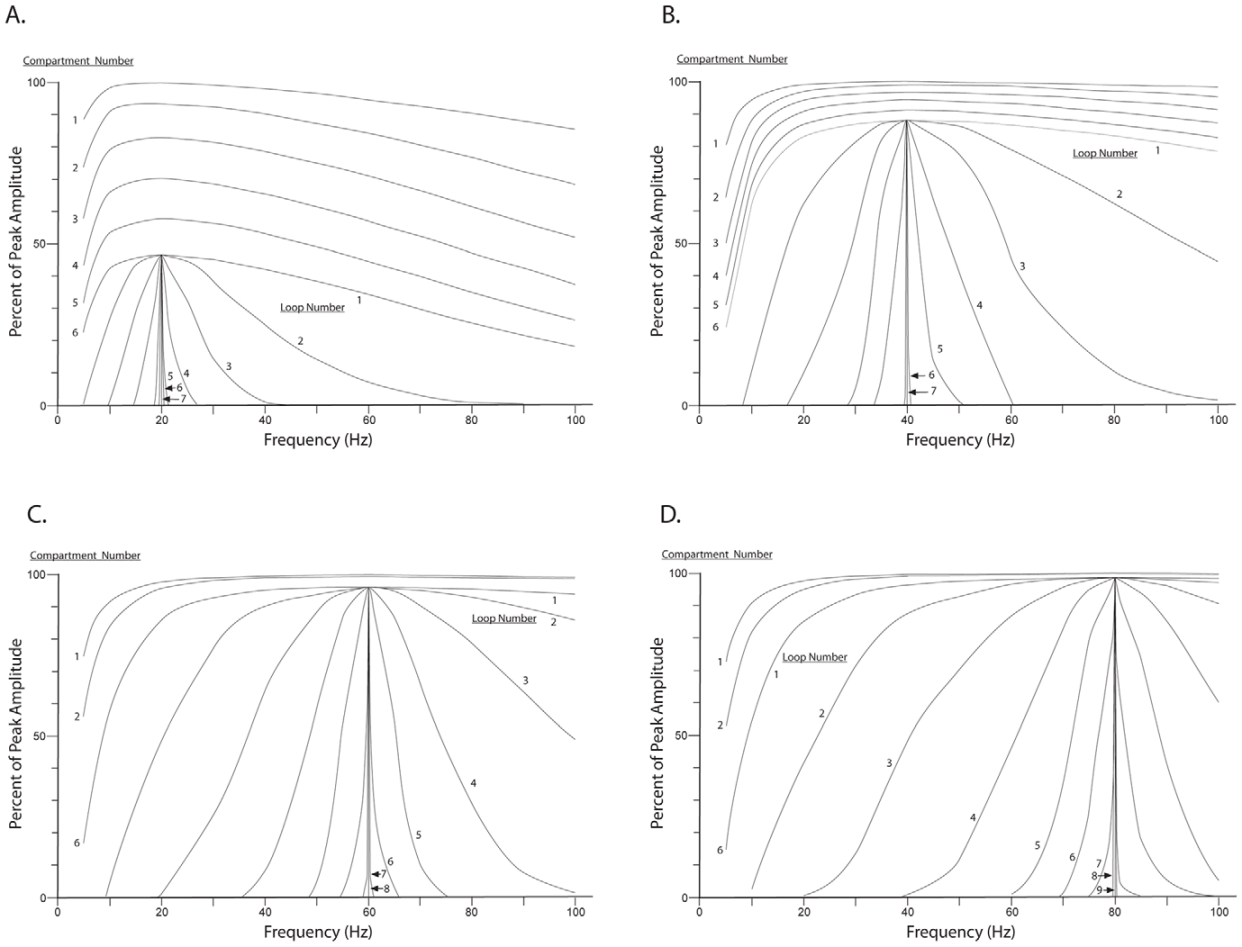
Narrowing of resonance curves. Resonance curves with peaks at 20, 40, 60, and 80 Hz, are narrowed by repeated cascades through 6 compartments via circuit loops through the thalamus. The resonance curves for the first 6 compartments are shown at the top of each of the 4 sets of curves (for clarity, the resonance curves for the 3rd, 4th, and 5th compartments are omitted for the 60 and 80 Hz conditions). The remaining curves in each of the 4 conditions show the effects of subsequent loops through the 6 compartments. The curve representing a loop is actually the output of the 6th compartment of each successive loop. Radial conductances = 105, 310, 627, and 1056 nS for the curves with peaks 20, 40, 60, and 80 Hz, respectively.

The magnitude of the impedance transfer functions of the cascade-loop model, plotted as percent of maximum voltage for 20, 40, 60, and 80 Hz peak frequencies, are shown in Figure 10. The results show two main tuning effects: The adjustment of the peak frequency of the impedance transfer function, shown here more definitively than in the single compartment model results of Figure 5, and the sharpening of the resonance curve around the peak frequency. The resonance curves corresponding to successive loops through the 6 compartments of an apical dendrite gradually narrows toward an asymptote described by a single vertical line. Visual inspection of the curve adjacent to the vertical line indicates that, at half the height of the curve, the width of the curve is approximately 1 Hz. It would seem that adding additional loops would narrow the width even further than the width obtained in the successions of curves observed in Figure 10. However, the presence of synaptic noise in the components of the cascade-loop model, particularly at linkages in the circuit loop, presumably opposes the narrowing process, and a balance between total synaptic noise and the compartmental narrowing process would then be expressed by a width value that conceivably could lie within a range of a fraction of a Hz to several tens of Hz.

According to the model, the peak frequency of the impedance transfer function is shifted upward or downward by varying the level of the total output conductance, which in turn is influenced by the tonic voltage level maintained inside the dendrite by the subthreshold synaptic activity at the hundreds of local spines. The area of the resonance curve around the peak is narrowed by successive electrical operations in the compartments through which the pulse travels as it makes its way toward the soma. As more compartments are traversed the resonance peak becomes more sharply defined.

## Discussion

### Resonance Tuning

Two aspects of resonance tuning are treated in this paper: (a) the selection of the peak frequency of the resonance curve, and (b) the sharpening of the resonance curve around the peak frequency.

(a) Selective activity is indicated by the finding that the impedance transfer function exhibits a peak, which suggests that only one compartment of the apical dendrite model is needed to exhibit some degree of selective filtering over the total range of frequencies of an injected input. Although the peak in the single-compartment case is weakly defined here, the peak becomes more noticeably narrowed when the compartment operations are repeated through more compartments. Thus when the current containing a range of frequencies is passed to the next compartment, the voltage of some frequencies will be larger than that of other frequencies. As mentioned in the previous section, the physical basis hypothesized for the selection of the (peak) resonance frequency is the outward conductance of potassium ions. A particular value of outward conductance is produced by a corresponding level of voltage on the inside surface of the membrane. This level of internal voltage is maintained by the ongoing subthreshold synaptic activity in local spines, which in turn is produced by pulses from axons of the local cortical circuitry. The local circuitry of the cortex is assumed to be noisy, owing to its complex circuitry and the firing characteristics of the constituent neurons. The source or sources that regulate the level of this background noise apparently are not yet known in detail. However, it would seem that the noise level for a specific minicolumn (or for a column cluster of minicolumns) should differ from the noise level of minicolumns serving other functions. For example, the ambient background noise level for minicolumns involved in processing the color red should exhibit a different noise level than the minicolumns involved in processing the square shape of an object. In this way the corticocortical circuits that connect Layer 2/3 pyramidal neurons of a group of minicolumns can function somewhat independently of the noise level of minicolumn local circuits, once the local noise level selects the resonance frequency of the participating pyramidal neurons. The amplitude of the noise of a particular minicolumn will, of course, exhibit a variance, and the size of this variance is expected to affect the sharpness of the resonance function.

(b) Sharpening activity, shown in Figure 10, is indicated by the increase in slope on each side of the resonance peaks of the curves as the number of participating compartments is increased. Thus it would appear that when cascades of electrical operations are performed across an appropriately chosen number of compartments (using the looping circuit to add compartments), the dendrite can exhibit a similar resonance curve, both in amplitude and in sharpness (often defined as 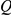), for any impedance peak in the range 10-100 Hz. However, when synaptic noise is taken into account, the predicted sharpness of a resonance curve will be attenuated. In general, the amount that noise attenuates the sharpness of the resonance peak depends upon the balance between number of compartments in the apical dendrite and the amount of noise in the corticothalamic loop.

In the search for experimental evidence supporting the existence and function of surface capacitance, 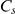, in our model we have analyzed the transfer impedance data shown in Figure 7 from the double-patching of neurons by Ulrich [45] from the viewpoint of classical electric network theory. The obtained impedance magnitude shape and zero phase angle condition would seem to require both low-pass and high-pass filtering effects that are only possible when the network model contains both series and shunt capacitances (assuming the absence of an inductance element). The series capacitance is defined as 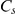 in our model. Future experiments that are designed to measure the complex transfer impedance (phase angle and magnitude) combined with known values of the membrane conductance and capacitance would enable a determination of the 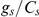 ratio in our model.

### The Effects of Synaptic Noise

While it is recognized that noise is added to the resonance processing within the pyramidal neuron itself, we assume here that the level of that noise is small relative to the level of noise added to the resonance processing by synapses. The issue of synaptic noise in the present model can be separated into the noise produced in two different places in the cascade-loop model of resonance tuning: the ambient noise in the thousands of spines along the apical dendrite shaft which produce subthreshold activation of neighboring conductance channels (chiefly potassium, in this model), and the noise at the synapses in the corticothalamic circuit which enables output current pulses from the dendritic compartments to loop back to the apical dendrite. Many hundreds of spines dot the apical dendrite (with a density of about one spine per dendritic length of 2 microns [33]), and the shapes of the spines apparently vary widely, without any clear indication of subtypes having specific functional properties [47]. Some spines apparently produce action potentials (from external axon contacts or from backpropagating action potentials) while others operate at subthreshold levels, which affect local conductance gating without producing action potentials. Whenever synaptic activity occurs in a spine it is expected that short-term depression and facilitation will follow and the balance between these two effects will serve as a transient context for the next synaptic event [16]. At present it is not known exactly how the operations underlying the tuning process in the dendrite may be affected by the transient context effect at the spines, including spines that produce subthreshold activity as well as those that produce suprathreshold spiking activity. One important consequence of altered synaptic context is that local conductances may be changed. In the present model of trains of current pulses, this contextual effect may be frequency dependent, and at higher frequencies, in particular, the effect may be repeated in a manner that prolongs the context over extended periods of time in which resonance is tuned.

The second location of synaptic noise of interest to the present model is in the part of the circuit loop that lies outside of the compartments of the dendrite. Included are synapses on the thalamic principal cell and on the inhibitory cells of the reticular nucleus (and synapses on the principal cell from the reticular nucleus and the interneurons), as well as the synapses of the principal cell axon on the apical dendrite of the cortical pyramidal cell. Additional synapses to be considered contact the corticothalamic loop from other sources. They include the important synapses by which ascending afferent fibers contact the thalamus en route to the primary sensory cortical areas. For the higher cortical areas, both ascending fibers from lower areas and descending fibers from frontal cortical areas make synaptic contacts with the thalamus (see Figure 8). Synaptic activities here initiate and help to maintain the loop activity over varying periods of time, The main synaptic activity is presumed to be the suprathreshold production of action potentials, and transient context effects are expected to influence spiking activity [15], [16]. However, it is not yet known the manner of and extent to which short-term resonance-like activities at these synapses influence the more global resonance tuning in the apical dendrite that is described by the present cascade-loop model.

If the traditional view of noise is applied to the workings of the present model, then all of the synapses under consideration, especially the synapses in the corticothalamic circuit, produce a significant level of noise that tends to flatten the shape of the resonance curve. While every cycle of the corticothalamic circuit adds noise to flatten the shape of the profile of the resonance curve, the next cascade through the apical dendrite compartments narrows the shape of the profile of the resonance curve. If the total noise in the corticothalamic loop is sufficiently large relative to the number of dendritic compartments, then the resonance curve cannot be narrowed to the maximum asymptotes shown in Figure 5. Therefore, a relatively large number of compartments in the apical dendrite provides a means of effectively offsetting the noise in the corticothalamic circuit. Hence, longer apical dendrites (e.g., in primates) that contain a larger number of compartments should produce more narrow resonance profiles, while very short apical dendrites (e.g., in mice) should show a limit on the narrowness of the resonance profile. For an illustrative comparison of apical dendrite lengths of Layer 5 and Layer 2/3 pyramidal neurons across 5 mammalian species see Figure 1 in [32].

## Methods

### Simulation method for a single compartment

The purpose of the single compartment model simulation is to demonstrate resonance in the apical dendrite by generating four resonance curves, corresponding to peak resonant frequencies of 20, 40, 60, and 80 Hz. To simulate the operations of a segment of the Layer 5 apical dendrite, we employ here the assumed equivalent circuit in a single compartment (see Figure 3). The choices of parameter values for the circuit are based on the following rationale: The diameter of the apical dendrite tapers from approximately 8.5 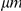 near the soma to a range of .30 to 1.33 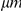 at the distal dendrite [33]. Here we select a dendritic segment lying in the middle region between the soma and distal endpoints, and we assume the dendrite diameter, 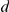, to have a constant value of 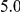 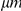 throughout the selected segment (following the compartment assumptions of [48] for the apical dendrite). The radial capacitance 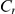 is assumed to be 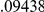 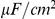, based on a diameter value of 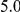 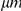, a compartment length of 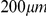, and a total membrane capacitance which is which is at the top of the range of capacitance values estimated by [25] for the Layer 5 apical dendrite. The relatively large capacitance value for 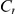 takes into account the presence of spines that increase the area of the membrane surface. The surface capacitance, 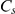, is frequency dependent and, following Equation (9), depends on the capacitance value of 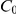 = 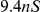, which is based on the minimum value of membrane capacitance given by [25]. The voltage-gated conductance, 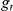, is estimated for each of the 4 resonance curves, corresponding to peak resonances of 20, 40, 60, and 80 Hz. The estimated 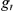 is assumed to be approximately constant over the the 6 compartments of the model, following the results of [25], who found that the voltage-gated 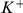 density gradient changes rapidly near the soma, while maintaining a relatively flat slope in the mid region of the apical dendrite. The time constant of the membrane, 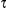, is assumed to be 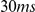, and the space constant, 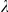, is frequency-dependent, with the steady state component, 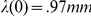 (based on a membrane resistance, 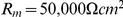, internal resistance, 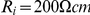, 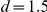 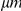). The frequency dependence of the space constant is defined as [36]:
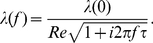
(14)


To calculate the transfer impedance, 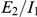, for the circuit of one compartment (see Figure 3), the expressions for 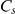 and 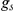, along with a value for the outward radial conductance, which is the sum of the voltage-gated conductance and the leak conductance, were condensed into the 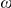 parameters and the constant 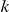 (via Equations (3–6). The 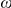 parameters and the constant 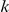 were then entered into Equation (12). The choice of the 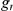 conductance value determines the location of the peak frequency and influences the shape of the transfer impedance curve plotted as a function of frequency. In particular, the maximum value, or peak, of the transfer impedance function will appear at a frequency that increases monotonically (but not linearly, owing to the frequency-dependent parameters) with 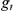.

### Simulation method for multiple compartments

We now consider the way that the multiple compartment model could tune an apical dendrite to narrow its resonance curve around a particular peak frequency. For the model, we select a segment of the apical dendrite 1200 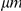 in length, which is assumed to have a constant diameter, 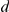 of 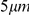. Each compartment is assumed to have a length of 200 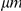, so that the total number of compartments of the model is 6. Following the work of [27], we assume that 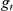 decreases linearly from the current injection site at the first compartment, and reaches a constant conductance level after the 6th compartment. Thereafter, a constant level of 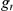 is assumed for each compartmental circuit.

The decrease in 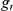 at each compartment is applied only to the 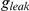 portion of the radial conductance. At the beginning of the first compartment the value of 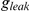 is assumed to be 30 nS (which is slightly larger than the 25 nS value assumed for 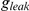 in the model of [20]), and this initial leak current is decreased by 5 nS to obtain the amount of leak current for the second compartment, which is 25 nS. After the 6th compartment the leak current is zero. Therefore,
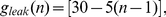
(15)


The excitatory input conductance to the nth compartment (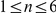) is denoted as 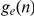, and it is related to the input of the previous compartment and to the leak conductance by the recursive equation
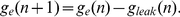
(16)


At the end of the first compartment, 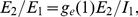
(17)


which states that the transfer impedance of that compartment circuit, 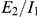, is multiplied by the output conductance of that compartment circuit, 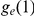, to obtain the voltage transfer function. For the second compartment output,
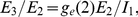
(18)


so that, in general,
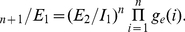
(19)


.The cascading operations across the 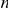 compartments is initiated by choosing a value for 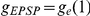. In order to plot the energy transfer values as a percentage of the maximum energy value, the value of 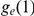 was calculated from the reciprocal of the peak impedance of each of the four curves, keeping in mind that the impedance is given at the end of the first compartment (which is the input to the second compartment):
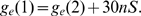
(20)


A cascade through the 6 compartments produces an array of new impedances across the frequency range. These impedance values are sent back to the first compartment via the corticothalamic loop (see Figure 8), and processed again through the 6 compartments. As a first approximation, the model assumes that no noise is added at synapses in the corticothalamic loop, nor within the pyramidal neuron itself. The impedance value at the peak frequency is assumed to be constant at the beginning of the first compartment across all loops, owing to the enhancement of the EPSP amplitude by the thalamic circuitry [49], [50]. Then the impedances of the other frequencies, above and below the peak frequency, are renormalized relative to the amplitude of the peak frequency. The looping sequence continues until the narrowing of the resonance curve reaches a width on the x-axis of approximately 1 Hz. Specifically, when the percentage values were less than 0.10 for each of the two frequencies bordering the peak frequency (e.g., for a peak frequency of 20 Hz, the bordering frequencies were 19 Hz and 21 Hz) the resonance curve was plotted as a single vertical line positioned at the peak frequency value.
